# Supplementary material for: Genetic risk factors for COVID-19 and influenza are largely distinct
Source: Nat Genet. 2024 Aug 5;56(8):1592–6. doi: 10.1038/s41588-024-01844-1 (PMC11319199; doi:10.1038/s41588-024-01844-1)
Supplement: Supplementary file 1 — Supplementary Note. [file 41588_2024_1844_MOESM1_ESM.pdf]

nature genetics

Supplementary information https://doi.org/10.1038/s41588-024-01844-1

Genetic risk factors for COVID-19 and influenza are largely distinct

In the format provided by the authors and unedited

SUPPLEMENTARY NOTE

Content

Association between the *ABO* locus and risk of influenza **2**

Association between the *B3GALT5* locus and risk of COVID-19 **6**

Genetic interaction effect between *ST6GAL1* and *B3GALT5* on risk of influenza **8**

***ADIPOQ* and *IGSF5*: two additional likely effector genes of influenza risk loci** **9**

Study limitations **10**

Phenome-wide association study of *ST6GAL1* and *B3GALT5* variants **12**

List of investigators from the Regeneron Genetics Center **15**

List of investigators from the Colorado Center for Personalized Medicine Biobank **18**

List of investigators from the Mayo Clinic Project Generation **19**

List of investigators from the UCLA ATLAS Consortium **21**

References **22**

Association between the ABO locus and risk of influenza

As highlighted in the main manuscript, among the 24 variants reported to associate with COVID-19 by the Host Genetics Initiative[2] (HGI; freeze 6), only one variant was associated with reported influenza infection after correcting for multiple testing (P<0.05/24=0.002): rs505922 in *ABO* (OR = 1.05 for the T allele, P=2.2x10^{-4}). This variant increased risk of reported influenza, while it decreased risk of COVID-19 (OR = 0.92).

A recent 23andMe study reported a similar pattern of opposing associations with COVID-19 and influenza at the *ABO* locus. Specifically, Shelton *et al.*[11] noted their lead COVID-19 variant at this locus (rs9411378:C, r^2=0.80 with the HGI variant rs505922) is in LD (r^2=0.57) with a frameshift variant in *ABO* (rs8176719) that determines the O blood group when present in the homozygous form. [We note that the HGI COVID-19 variant rs505922 is in higher LD with the *ABO* frameshift variant, r^2=0.89] Shelton et al. then showed that individuals with O blood group have lower risk of COVID-19 (OR~0.8) but higher risk of influenza, for example an OR=1.05 (P=1.8x10^{-6}) for association with influenza in the 2017 season. Importantly, the associations with influenza in the 23andMe study[11] (but not in AncestryDNA) were based on information collected prior to the COVID-19 pandemic. Despite this, Shelton et al. suggested that the association between *ABO* and COVID-19 could have resulted from a subtle form of collider bias (but no additional information provided).

We considered a few possibilities to explain the apparent inconsistent association at the *ABO* locus between COVID-19 and influenza. First, the association between the *ABO* locus and influenza may be a false-positive finding: neither the blood group association reported by 23andMe nor results from our influenza GWAS reach genome-wide significance.

Second, if the association between the *ABO* locus and influenza is a true-positive finding, it may represent a genetic signal distinct from the COVID-19 association. Consistent with this possibility, we found that the variant most strongly associated with influenza risk in our meta-analysis of discovery and replication GWAS (rs2519093, OR=1.05, P=1.4x10^{-6}) is (i) not in high LD with the HGI COVID-19 variant rs505922 (r^2=0.43), suggesting that it is not the same signal; (ii) not in high LD with any of the three variants that define ABO blood groups, namely the frameshift variant rs8176719 (r^2=0.31), rs8176747 (r^2=0.01) and rs41302905 (r^2=0.01), indicating that the association with influenza is unlikely to be mediated by variation in ABO blood groups; and (iii) in high LD (r^2>0.80) with variants previously reported to associate with the viral-related diseases tonsillectomy[31], gastrointestinal infections[32] and childhood ear infections[31] (see **Figure S1** below), as well as lipid-related measurements[33], stroke[34], granulocyte counts[35], allergic disease[36] and C-reactive protein levels[37], among others. These results are consistent with the notion that the influenza and COVID-19 genetic signals at the *ABO* locus are distinct, with the former being shared with many other diseases and traits.

Supplementary Note Figure 1. Detailed association results between common variants at the *ABO* locus and influenza risk in the meta-analysis of AncestryDNA and biobank cohorts.

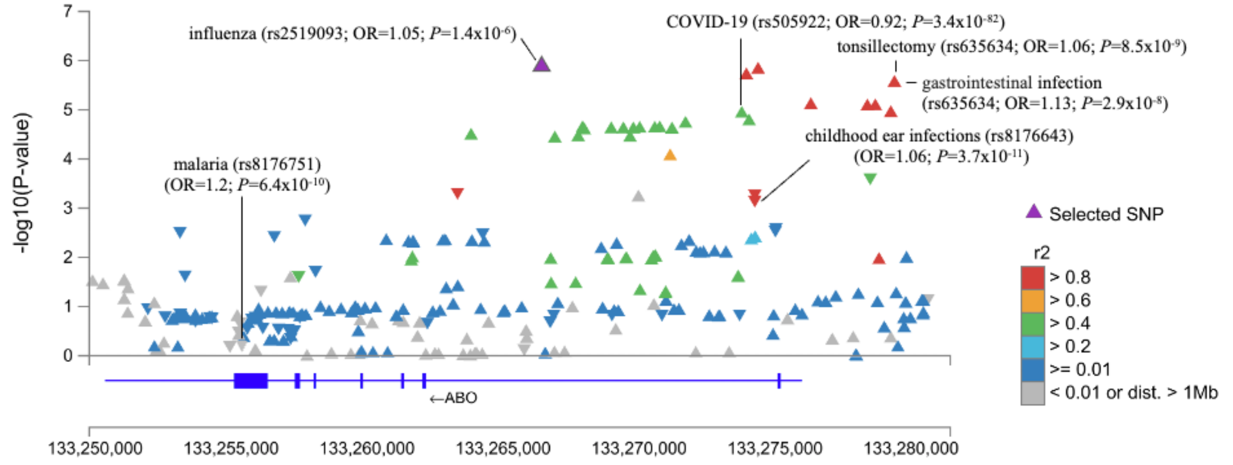

Upward facing triangles represent variants with an odds ratio (OR)>1, and downward facing

triangles represent OR<1. Unadjusted *P*-values derived from Firth-regression (two-sided test) implemented in REGENIE[8].

Third, we observed that COVID-19 cases were enriched among influenza controls in the AncestryDNA cohort (see **Supplementary Note Table 1** below). As such, we hypothesized that this bias could also contribute to the opposing association at the *ABO* locus for COVID-19 and influenza.

Supplementary Note Table 1. Prevalence of a positive test for COVID-19 among individuals with and without influenza in GWAS cohorts.

Cohort	N influenza cases (% with COVID-19)	N influenza controls (% with COVID-19)
AncestryDNA	18334 (6.69%)	276295 (11.64%)
UKB, GHS, PMBB, UCLA, Mayo Clinic, Colorado	14442 (10.98%)	844650 (5.73%)

To address this possibility, we repeated the association between the COVID-19 risk variant rs505922 and influenza in the AncestryDNA cohort after adjusting for COVID-19 status. In this analysis, the association between rs505922 and self-reported influenza was attenuated and only borderline significant (OR = 1.027, *P*=0.07). These results indicate that the enrichment of COVID-19 cases among influenza controls in the AncestryDNA cohort led to a biased effect size estimate for influenza for this variant. In contrast, we did not observe an inverse association between COVID-19 and influenza status in the biobank cohorts (**Supplementary Note Table 1**); in a meta-analysis of GWAS from these cohorts, the association between rs505922 and influenza (OR=1.027, *P*=0.0084) had the same magnitude of effect as observed in AncestryDNA after adjusting for COVID-19 status.

Overall, these findings suggest that there may be a weak association between the ABO locus and influenza risk, but that this is unlikely to be the same genetic signal observed for COVID-19.

Association between the *B3GALT5* locus and risk of COVID-19

Having identified and replicated two GWAS loci for influenza (in *ST6GAL1* and *B3GALT5*), we inquired if either were associated with COVID-19, based on the published HGI multi-ancestry meta-analysis for reported infection, risk of hospitalization or risk of severe disease (defined by respiratory failure or death)[2]. After Bonferroni correction (P<0.05/6=0.0083), we found a modest association between rs2837113 in *B3GALT5* and risk of SARS-CoV-2 infection (OR=1.014, 95% CI 1.006 to 1.022, P=8.9x10^{-4}; **Supplementary Table 4**).

However, as observed for *ABO*, the direction of effect of rs2837113 on COVID-19 was opposite that of influenza, which could potentially arise if there was an inverse phenotypic association between the two conditions in the contributing HGI cohorts, as we observed for AncestryDNA (specifically, influenza controls, who are more likely to carry the rs2837113:A influenza-protective allele, were enriched among COVID-19 cases; Supplementary Note **Table 1**). To test this possibility at least within our own data, we compared the association between rs2837113 and risk of SARS-CoV-2 infection in AncestryDNA, before and after adjusting for self-reported influenza status. We found that the association was slightly attenuated after adjusting for influenza status (OR=1.018 before vs. OR=1.010 after adjustment; **Supplementary Note Table 2**). Although inconclusive, these results raise the possibility that the association between rs2837113 and SARS-CoV-2 infection may be inflated if influenza controls were enriched among COVID-19 cases studied by the HGI.

Supplementary Note Table 2. Association between the *B3GALT5* variant identified in the GWAS of self-reported influenza and risk of SARS-CoV-2 infection in the AncestryDNA cohort, before and after adjusting for influenza status.

Variant, rsID, nearest gene	Association with COVID-19 positive vs negative or unknown	Odds Ratio* (95% CI)	*P*-value	Alternate Allele frequency	N cases	N controls
21:39676197:G:A, rs2837113, B3GALT5	Before adjusting for influenza status	1.018 (1.000,1.036)	0.0519	0.509	33603	191189
	After adjusting for influenza status	1.010 (0.992, 1.028)	0.2636	0.510	33396	190079

Unadjusted *P*-values derived from Firth-regression (two-sided test) implemented in REGENIE[8]. *

Effect allele: A

Genetic interaction effect between *ST6GAL1* and *B3GALT5* variants on risk of influenza

Given that both *ST6GAL1* (adds sialic acid to a galactose) and *B3GALT5* (adds a galactose to a GlcNAc) play a role glycan structure synthesis, we tested for a genetic interaction effect (i.e., epistasis) between both loci, specifically between variants rs13322149 (*ST6GAL1*) and rs2837113 (*B3GALT5*). Such an interaction effect could arise if, for example, a lower rate of sialic acid addition to galactose (due to the *ST6GAL1* polymorphism) resulted in stronger protection from influenza in individuals with a higher rate of galactose addition to a GlcNAc (due to the *B3GALT5* polymorphism). To test for epistasis between the two loci, we estimated the SNPxSNP interaction effect on risk of influenza separately in AncestryDNA and six biobanks with available individual-level data (Colorado, DiscovEHR, Mayo Clinic, PMBB, UCLA, and UKB), for a combined sample size of 32,776 cases and 1,120,945 controls. After meta-analyzing the estimated interaction effect across all cohorts, we found no evidence for epistasis between the two loci (interaction OR=1.027, 95% CI 0.975 to 1.081, *P*=0.32).

***ADIPOQ* and *IGSF5*: two additional likely effector genes of influenza risk loci**

Four genes were prioritized as likely effector genes underlying the GWAS signals identified for influenza (**Supplementary Tables 7** and **8**): *ST6GAL1* and *ADIPOQ* at the 3q27.3 locus, and *B3GALT5* and *IGSF5* at the 21q22.2 locus. *ST6GAL1* and *B3GALT5* were the nearest genes to the GWAS lead variant at each locus and are discussed in more detail in the main text. *ADIPOQ* and *IGSF5* are briefly discussed here.

ADIPOQ: this gene encodes adiponectin, a hormone secreted by adipocytes that regulates energy homeostasis, glucose and lipid metabolism[38]. *ADIPOQ* was the second nearest gene to the lead influenza variant (121 kb upstream of rs13322149); it was identified as a likely effector gene of the GWAS signal because rs13322149 was in high LD (r^2>0.8) with variants located in putative enhancers for *ADIPOQ* in B cells[39] (**Supplementary Table 8)**. Related to this, rs13322149 was also in high LD with a variant reported to associate with plasma adiponectin levels (rs73187787, r^2=0.95)[40]; the rs13322149:T allele that lowers risk of influenza is in phase with the rs73187787:T allele that increases adiponectin levels.

IGSF5: this gene encodes an immunoglobulin-type cell adhesion molecule with relatively low expression across most tissues studied by GTEx[41]. *IGSF5* was the second nearest gene (68 kb downstream) to the lead influenza variant (rs2837113), with the following data suggesting it is a likely effector gene underlying the GWAS signal. First, a sentinel eQTL for *IGSF5* in skin tissue studied by GTEx (rs7278671) is in high LD with the lead influenza variant rs2837113 (r^2=0.89); the rs2837113:A allele that lowers risk of influenza is in phase with the rs7278671:G allele that increases *IGSF5* expression in the skin (**Supplementary Table 7**). Second, in our exome sequencing study, a rare missense variant in *IGSF5* was associated with higher risk of medical record influenza (**Supplementary Table 9**).

Study limitations

The following limitations should be considered when interpreting results from our study.

Phenotype misclassification. There may be some level of phenotype misclassification for both cases and controls. The control group in the discovery and replication influenza GWAS may have included individuals (i) infected with influenza but that were asymptomatic or never tested; and (ii) who were not exposed to the influenza virus (some of which tested negative). The latter is likely to have been more common during the COVID-19 pandemic, due to public health policies (e.g. use of masks, social distancing) that limited exposure to airborne viruses. Similarly, the case group in the influenza infection GWAS was likely heterogeneous (e.g., included a range of disease severity). As a result, it is not straightforward to interpret genetic associations with these infection phenotypes, as they could capture multiple dimensions of susceptibility, for example risk of infection once exposed or risk of severe outcomes once infected. We addressed this limitation by performing sensitivity analyses which overall showed that both influenza loci remained associated across a range of additional phenotypes, from looser (flu-like symptoms) to stricter (positive viral culture) definitions of infection. Furthermore, both loci were associated with lower risk of hospitalization among infected cases, significantly so for the *B3GALT5* variant.

Potential confounding effects of unmeasured risk factors for influenza infection. The inclusion of misspecified controls reduces power to detect true associations and, in some situations (for example, when a variant is strongly associated with risk of virus exposure), can also increase the risk of false-positive associations with risk of infection after exposure, as previously noted[42]. To address the latter possibility (i.e. could the *ST6GAL1* and *B3GALT5* variants be associated with an outcome that is a risk factor for exposure to influenza?), we performed a phenome-wide association study for each variant but found no associated outcomes (detailed in the subsequent

section). As such, it is unlikely that the associations we describe for influenza arise from the confounding effect of a correlated outcome.

Other notable caveats that limit the generalizability of our results include (ii) the use of self-reported influenza information (with its inherent limitations) in the AncestryDNA cohort, which is self-selected, older, more European and more female than the broader US population, as noted previously[43] (iii) undetermined influenza strain infecting GWAS participants; and (iv) having severity outcomes available in only two cohorts (GHS and UKB).

Phenome-wide association study of *ST6GAL1* and *B3GALT5* variants

Recently, Duchen et al. [42] investigated the impact of using population-level controls on the rate of false-positive associations with infectious diseases. Specifically, the authors made the following major observations:

- Using simulated data, the authors found that when a variant X affects risk of exposure to a pathogen but not risk of infection once exposed, then:
    - Using population-level controls (as opposed to individuals exposed but without symptomatic infection) can sometimes yield a spurious association between variant X and risk of infection (see their Figure 2)
    - Intuitively, this makes sense: 100% of individuals with symptomatic infection had a previous pathogen exposure, whereas that is unlikely to be the case for 100% of population-level controls. In other words, the outcome that is determined by variant X (risk of exposure) will often be more common in individuals with symptomatic infection than in population-level controls.
- The probability that variant X will show a spurious association with risk of infection when using population-level controls decreases with increasing prevalence of pathogen exposure (see their Table 2).
    - This also makes sense intuitively: at the extreme, when the prevalence of pathogen exposure is 100%, the outcome that is determined by variant X (risk of exposure) has the same frequency in both the case and control groups, and so no association is observed with variant X.
- Using real world data, the authors compared results between two GWAS that used the same set of cases but different control groups:

- GWAS #1: 702 individuals infected with hepatitis C virus (HCV) but who cleared the virus (source: HCV consortium) vs. 1,037 individuals with persistent HCV infection (source: HCV consortium). Two loci discovered at $P<5x10^{-8}$: *HLA* and *IFNL3*.
- GWAS #2: 702 individuals infected with hepatitis C virus (HCV) but who cleared the virus (source: HCV consortium) vs. 370K ancestry-matched population-level controls with unknown HCV status (source: UKB). This GWAS identifies the same two loci discovered in GWAS #1 (*HLA*, *IFNL3*) and two additional loci at $P<5x10^{-8}$: *STX18* and a locus on chromosome 2.

- Additional analyses suggest that one of the novel associations discovered in GWAS #2 (*STX18*) is an example of a locus that affects risk of exposure to HCV and not risk of HCV clearance. Specifically, the authors suggest that the *STX18* variant increases risk of hemophilia, which in turn increases risk of exposure to HCV. This conclusion is based on the following observations:
  - The signal was attenuated (but not fully eliminated) after excluding individuals with hemophilia.
  - The signal was considerably attenuated and not significant in GWAS #1;
  - The signal was stronger when comparing all HCV cases (with cleared or persistent infection) against UKB population-level controls.

If the type of bias described by Duchen et al. explained the two associations we discovered for influenza (*ST6GAL1* and *B3GALT5*), then these loci would have to be strongly associated with an outcome that increased the risk of exposure to the influenza virus.

It is not obvious what heritable outcome(s) might strongly increase risk of exposure to the influenza virus and that could potentially explain the flu association with *ST6GAL1* and *B3GALT5*. Nonetheless, to address this possibility, we performed a phenome-wide association study (PheWAS) for the *ST6GAL1* and *B3GALT5* variants in the UKB (N=450K) and GHS (N=175K) studies, testing each variant for association with 6,221 binary and 2,916 quantitative outcomes. We found no outcomes associated with either variant at a $P<0.05/(2 \text{ variants x } 9137 \text{ traits tested}) = 2.68 \times 10^{-6}$, indicating that these variants do not have a strong effect on any outcome measured in these studies. As such, we think it is highly unlikely that the associations we describe for influenza arise from the confounding effect of a correlated outcome.

List of investigators from the Regeneron Genetics Center

All authors are listed in alphabetical order.

RGC Management and Leadership Team

Gonçalo Abecasis, D.Phil. [1], Aris Baras, M.D. [1], Michael Cantor, M.D. [1], Giovanni Coppola, M.D. [1], Andrew Deubler[1], Aris Economides, Ph.D. [1], Katia Karalis, Ph.D. [1], Luca A. Lotta, M.D., Ph.D. [1], John D. Overton, Ph.D. [1], Jeffrey G. Reid, Ph.D. [1], Katherine Siminovitch, M.D. [1], Alan Shuldiner, M.D. [1]

Sequencing and Lab Operations

Christina Beechert[1], Caitlin Forsythe, M.S. [1], Erin D. Fuller [1], Zhenhua Gu, M.S. [1], Michael Lattari [1], Alexander Lopez, M.S. [1], John D. Overton, Ph.D. [1], Maria Sotiropoulos Padilla, M.S. [1], Manasi Pradhan, M.S. [1], Kia Manoochehri, B.S. [1], Thomas D. Schleicher, M.S. [1], Louis Widom [1], Sarah E. Wolf, M.S. [1], Ricardo H. Ulloa, B.S. [1]

Clinical Informatics

Amelia Averitt, Ph.D. [1], Nilanjana Banerjee, Ph.D. [1], Michael Cantor, M.D. [1], Dadong Li, Ph.D. [1], Sameer Malhotra, M.D. [1], Deepika Sharma, MHI [1], Jeffrey Staples, Ph.D. [1]

Genome Informatics

Xiaodong Bai, Ph.D. [1], Suganthi Balasubramanian, Ph.D. [1], Suying Bao, Ph.D. [1], Boris Boutkov, Ph.D. [1], Gisu Eom, B.S. [1], Lukas Habegger, Ph.D. [1], Alicia Hawes, B.S. [1], Olga Krasheninina, M.S. [1], Rouel Lanche, B.S. [1], Adam J. Mansfield, B.A. [1], Evan K. Maxwell, Ph.D. [1], George

Mitra, B.A. [1], Mona Nafde, M.S. [1], Sean O'Keeffe, Ph.D. [1], Max Orelus, B.B.A. [1], Razvan Panea, Ph.D. [1], Tommy Polanco, B.A. [1], Ayesha Rasool, M.S. [1], Jeffrey G. Reid, Ph.D. [1], William Salerno, Ph.D. [1], Kathie Sun, Ph.D. [1], Jiwen Xin, Ph.D. [1]

Analytical Genomics and Data Science

Gonçalo Abecasis, D.Phil. [1], Joshua Backman, Ph.D. [1], Amy Damask, Ph.D. [1], Manuel Allen Revez Ferreira, Ph.D. [1], Arkopravo Ghosh, M.S. [1], Christopher Gillies, Ph.D. [1], Lauren Gurski, B.S. [1], Eric Jorgenson, Ph.D. [1], Michael Kessler, Ph.D. [1], Jack Kosmicki, Ph.D. [1], Nan Lin, Ph.D. [1], Adam Locke, Ph.D. [1], Jonathan Marchini, Ph.D. [1], Anthony Marcketta, M.S. [1], Joelle Mbatchou, Ph.D. [1], Arden Moscati, Ph.D. [1], Priyanka Nakka, Ph.D. [1], Charles Paulding, Ph.D. [1], Carlo Sidore, Ph.D. [1], Eli Stahl, Ph.D. [1], Kyoko Watanabe, Ph.D. [1], Bin Ye, Ph.D. [1], Blair Zhang, Ph.D. [1], Andrey Ziyatdinov, Ph.D. [1]

Therapeutic Area Genetics

Parsa Akbari, Ph.D. [1], Ariane Ayer, B.S. [1], Jonas Bovijn M.D. [1], Giovanni Coppola, M.D. [1], Tanima De, Ph.D. [1], Sahar Gelfman, Ph.D. [1], Sujit Gokhale, B.E. [1], Aysegul Guvenek, Ph.D. [1], Mary Haas, Ph.D. [1], George Hindy, Ph.D. [1], Manav Kapoor, Ph.D. [1], Luca A. Lotta, M.D. [1], Veera Rajagopal, Ph.D. [1], Moeen Riaz , Ph.D. [1], Juan Rodriguez-Flores, Ph.D. [1,] Alan Shuldiner, M.D. [1], Katherine Siminovitch, M.D. [1], Olukayode Sosina, Ph.D. [1], Gannie Tzoneva, Ph.D. [1], Niek Verweij, Ph.D. [1], Bin Ye, Ph.D. [1]

RGC Biology

Shek Man Chim, Ph.D. [1], Valerio Donato, Ph.D. [1], Aris Economides, Ph.D. [1], Daniel Fernandez, M.S. [1], Giusy Della Gatta, Ph.D. [1], Alessandro Di Gioia, Ph.D. [1], Kristen Howell, M.S. [1], Katia Karalis, Ph.D. [1], Lori Khrimian, Ph.D. [1], Minhee Kim, Ph.D. [1], Hector Martinez[1], Lawrence Miloscio, B.S. [1], Sheilyn Nunez, B.S. [1], Elias Pavlopoulos, Ph.D. [1], Trikaldarshi Persaud, B.S. [1]

Research Program Management & Strategic Initiatives

Esteban Chen, M.S. [1], Marcus B. Jones, Ph.D. [1], Michelle G. LeBlanc, Ph.D. [1], Jason Mighty, Ph.D. [1], Lyndon J. Mitnaul, Ph.D. [1], Nirupama Nishtala, Ph.D. [1], Nadia Rana, Ph.D. [1]

Affiliations:

1. Regeneron Genetics Center, Tarrytown, NY USA

List of investigators from the Colorado Center for Personalized Medicine Biobank

All authors are listed in alphabetical order.

Christina L. Aquilante[5], Lisen Axell[5], Kathleen C. Barnes[5], Ian M. Brooks[5], Tonya M. Brunetti[5], Emily Casteel[5], Sameer Chavan[5], Marilyn Coors[5], Kristy Crooks[5], Michelle N. Edelmann[5], Matthew Fisher[5], Debashis Ghosh[5], Christopher R. Gignoux[5], Casey S. Greene[5], Emily Hearst[5], Steve Hess[5], Madelyne Hull[5], Jean Jirikowic[5], Michael G. Kahn[5], David Kao[5], Lisa Ku[5], Elizabeth Kudron[5], Yee Ming Lee[5], Joseph Lesny[5], Meng Lin[5], Jan Lowery[5], Rasika Mathias[5], David A. Mayer[5], Ethan Moore[5], Joshua L. Morgenstern[5], J. Tacker Patton[5], Brett Peterson[5], Tzu L. Phang[5], Nikita Pozdeyev[5], Nicholas Rafaels[5], Emily R. Roberts[5], Alanna N. Roff[5], Elise L. Shalowitz[5], Jonathan A Shortt[5], Harry Smith[5], Adrian Stewart[5], Anna Tanaka[5], Matthew R.G. Taylor[5], Emily Todd[5], Sarah White[5], Stephen J. Wicks[5], Laura K. Wiley[5], Cole Williams, Melissa P. Wilson[5], Richard Zane[5]

[5]Colorado Center for Personalized Medicine, Aurora, CO, USA

List of investigators from the Mayo Clinic Project Generation

All authors are listed in alphabetical order.

PG Leadership Team

James R. Cerhan, M.D.[6]; Fergus J. Couch, Ph.D.[6]

Statistical Genetics and Bioinformatics

Nicholas B. Larson, Ph.D., M.S.[6]; Eric W. Klee, Ph.D.[6]; Zachary S. Fredericksen, M.S.[6]; Steven N. Hart, Ph.D.[6]; Kimberly P. Lauer, M.S.[6]

Clinical Informatics

Hongfang Liu, Ph.D.[6]; Andrew Wen, M.S.[6]

Laboratory Operations

Mine Cicek, Ph.D.[6]

Registry Principal Investigators

J. Eric Ahlskog, M.D., Ph.D.[6]; Samuel O. Antwi, Ph.D.[6]; Andrew D. Badley, M.D.[6]; Jamie N. Bakkum-Gamez, M.D.[6]; Suzette J. Bielinski, Ph.D., M.Ed.[6]; Joanna M. Biernacka, Ph.D.[6]; Lisa A. Boardman, M.D.[6]; James H. Bower, M.D.[6]; James R. Cerhan, M.D., Ph.D.[6]; Mine Cicek, Ph.D.[6]; Fergus J. Couch, Ph.D.[6]; Angela Dispenzieri, M.D.[6]; Sean C. Dowdy, M.D.[6]; Jeanette E. Eckel Passow, Ph.D.[6]; Gretchen E. Glaser, M.D.[6]; Ellen L. Goode, Ph.D.[6]; Peter C. Harris,

Ph.D.[6]; W. Michael Hooten, M.D.[6]; Robert B. Jenkins, M.D., Ph.D.[6]; Scott H. Kaufmann, M.D., Ph.D.[6]; Richard B. Kennedy, Ph.D.[6]; Iftikhar J. Kullo, M.D.[6]; John C. Lieske, M.D.[6]; Minetta C. Liu, M.D.[6]; Daniel J. Ma, M.D.[6]; Sarah A. McLaughlin, M.D.[6]; Robert W. Mutter, M.D.[6]; Janet E. Olson, Ph.D[6].; Naveen L. Pereira, M.D.[6]; Vijay K. Ramanan, M.D., Ph.D.[6]; Celine M. Vachon, Ph.D.[6]; Prashanthi Vemuri, Ph.D.[6]; Ping Yang, M.D., Ph.D.[6]

Management

Andrew J. Danielsen, M.S.[6]; Jonathan J. Harrington, B.A.[6]; Jennifer M. Kushwaha[6]

[6]Mayo Clinic, Rochester, MN, USA

List of investigators from the University of California Los Angeles ATLAS Consortium

All authors are listed in alphabetical order.

Maryam Ariannejad[7], Joycee J. Berin[7], Yael Berkovich[7], Joseph DeYoung[7], Daniel H. Geschwind[7], So-Young Hong[7], Clara M. Lajonchere[7], Clara Magyar[7], Marissa Mercado[7], Ghouse Mohammed[7], Bogdan Pasaniuc[7], Jay Shah[7], Dawn C. Ward[7]

[7]Institute of Precision Health, University of California, Los Angeles, Los Angeles, CA, USA

References

31. Tian, C. *et al.* Genome-wide association and HLA region fine-mapping studies identify susceptibility loci for multiple common infections. *Nature Communications* **8**, 599 (2017).
32. Nudel, R. *et al.* A large population-based investigation into the genetics of susceptibility to gastrointestinal infections and the link between gastrointestinal infections and mental illness. *Hum Genet* **139**, 593-604 (2020).
33. Richardson, T.G. *et al.* Characterising metabolomic signatures of lipid-modifying therapies through drug target mendelian randomisation. *PLoS Biol* **20**, e3001547 (2022).
34. Malik, R. *et al.* Multiancestry genome-wide association study of 520,000 subjects identifies 32 loci associated with stroke and stroke subtypes. *Nat Genet* **50**, 524-537 (2018).
35. Astle, W.J. *et al.* The Allelic Landscape of Human Blood Cell Trait Variation and Links to Common Complex Disease. *Cell* **167**, 1415-1429.e19 (2016).
36. Ferreira, M.A.R. *et al.* Age-of-onset information helps identify 76 genetic variants associated with allergic disease. *PLoS Genet* **16**, e1008725 (2020).
37. Han, X. *et al.* Using Mendelian randomization to evaluate the causal relationship between serum C-reactive protein levels and age-related macular degeneration. *Eur J Epidemiol* **35**, 139-146 (2020).
38. Achari, A.E. & Jain, S.K. Adiponectin, a Therapeutic Target for Obesity, Diabetes, and Endothelial Dysfunction. *Int J Mol Sci* **18**(2017).
39. Nasser, J. *et al.* Genome-wide enhancer maps link risk variants to disease genes. *Nature* **593**, 238-243 (2021).
40. Spracklen, C.N. *et al.* Adiponectin GWAS loci harboring extensive allelic heterogeneity exhibit distinct molecular consequences. *PLoS Genet* **16**, e1009019 (2020).
41. Consortium, G.T. The Genotype-Tissue Expression (GTEx) project. *Nat Genet* **45**, 580-5 (2013).
42. Duchen, D. *et al.* Pathogen exposure misclassification can bias association signals in GWAS of infectious diseases when using population-based common control subjects. *Am J Hum Genet* **110**, 336-348 (2023).
43. Knight, S.C. *et al.* COVID-19 susceptibility and severity risks in a cross-sectional survey of over 500 000 US adults. *BMJ Open* **12**, e049657 (2022).
