# Supplementary material for: Genetic risk factors for COVID-19 and influenza are largely distinct
Source: Nat Genet. 2024 Aug 5;56(8):1592–6. doi: 10.1038/s41588-024-01844-1 (PMC11319199; doi:10.1038/s41588-024-01844-1)
Supplement: Supplementary file 2 — Reporting Summary [file 41588_2024_1844_MOESM2_ESM.pdf]

nature portfolio

Corresponding author(s): M.A.R.F

Last updated by author(s): Feb 7, 2024

Reporting Summary

Nature Portfolio wishes to improve the reproducibility of the work that we publish. This form provides structure for consistency and transparency in reporting. For further information on Nature Portfolio policies, see our Editorial Policies and the Editorial Policy Checklist.

Statistics

For all statistical analyses, confirm that the following items are present in the figure legend, table legend, main text, or Methods section.

n/a	Confirmed	
☐	☒	The exact sample size (*n*) for each experimental group/condition, given as a discrete number and unit of measurement
☐	☒	A statement on whether measurements were taken from distinct samples or whether the same sample was measured repeatedly
☐	☒	The statistical test(s) used AND whether they are one- or two-sided<br>*Only common tests should be described solely by name; describe more complex techniques in the Methods section.*
☐	☒	A description of all covariates tested
☐	☒	A description of any assumptions or corrections, such as tests of normality and adjustment for multiple comparisons
☐	☒	A full description of the statistical parameters including central tendency (e.g. means) or other basic estimates (e.g. regression coefficient) AND variation (e.g. standard deviation) or associated estimates of uncertainty (e.g. confidence intervals)
☐	☒	For null hypothesis testing, the test statistic (e.g. *F*, *t*, *r*) with confidence intervals, effect sizes, degrees of freedom and *P* value noted<br>*Give P values as exact values whenever suitable.*
☒	☐	For Bayesian analysis, information on the choice of priors and Markov chain Monte Carlo settings
☒	☐	For hierarchical and complex designs, identification of the appropriate level for tests and full reporting of outcomes
☐	☒	Estimates of effect sizes (e.g. Cohen's *d*, Pearson's *r*), indicating how they were calculated

Our web collection on statistics for biologists contains articles on many of the points above.

Software and code

Policy information about availability of computer code

Data collection

Individual-level exome sequencing, genotype and phenotype data is available to approved researchers via UKB at: https://www.ukbiobank.ac.uk/enable-your-research. Influenza GWAS summary statistics from FinnGen Release 8 are available to approved individuals after accepting the terms and licenses of the data. Influenza A seropositivity from Scepanovic et al., were downloaded from the GWAS catalogue (accession #GCST006339).

Single-sample processing, all in DNAnexus
-Conversion of sequencing data in BCL format to FASTQ format and the assignments of paired-end sequence reads to samples based on 10-base barcodes; bcl2fastq v2.19.0 https://support.illumina.com/sequencing/sequencing_software/bcl2fastq-conversion-software.html
-Read alignment; bwa 0.7.17 http://bio-bwa.sourceforge.net
-Duplicate marking, stats gathering; picard v1.141 https://broadinstitute.github.io/picard/
-SAM/BAM/CRAM file generation and manipulation; samtools v1.7 http://www.htslib.org
-Variant calling; WeCall v1.1.2 https://github.com/Genomicsplc/wecall
-Sequence Quality Control; FastQC 0.11.8 http://www.bioinformatics/babraham.ac.uk/projects/fastqc/
-VCF file manipulation and index generation; bcftools v1.7 http://www.htslib.org, bgzip/tabix v1.7 http://www.htslib.org
-haplotyping (Ancestry.com); Eagle v2.4.1 https://github.com/poruloh/Eagle
-imputation (Ancestry.com): Minimac4 v1.01 https://github.com/statgen/Minimac4

Generation of "freeze" data
-Joint genotyping to generate project-level VCF (pVCF) files; GLnexus v1.4.5 https://github.com/dnanexus-rnd/GLnexus
-Generation of variant representations in PLINK format; PLINK v1.90b6.21 https://www.cog-genomics.org/plink2/

	-Ancestry predictions, IBD (Identity-by-descent) estimate, and pedigree reconstruction; PLINK v1.90b6.21 https://www.coggenomics.org/plink2/
Data analysis	- association testing: REGENIE v3.1.3 https://github.com/rgcgithub/regenie.<br>- meta-analysis: METAL (2020-05-05) https://github.com/statgen/METAL.<br>- various: python v3.8 https://www.python.org/downloads/; R v4.0.4 https://cran.r-project.org, R packages include ggplot2 (v3.4.2) and patchwork (v1.1.3). Python packages include pandas (v2.0.3) and numpy (v1.25.2).<br>- Plots for in vitro experiments: GraphPad Prism 9.3.0<br>- qPCR analysis: QuantStudio 6 (v2.6)

For manuscripts utilizing custom algorithms or software that are central to the research but not yet described in published literature, software must be made available to editors and reviewers. We strongly encourage code deposition in a community repository (e.g. GitHub). See the Nature Portfolio guidelines for submitting code & software for further information.

Data

Policy information about availability of data

All manuscripts must include a data availability statement. This statement should provide the following information, where applicable:

- Accession codes, unique identifiers, or web links for publicly available datasets
- A description of any restrictions on data availability
- For clinical datasets or third party data, please ensure that the statement adheres to our policy

Summary statistics from our GWAS will be made publicly available. Individual-level exome sequencing, genotype and phenotype data is available to approved researchers via UKB at: https://www.ukbiobank.ac.uk/enable-your-research. FinnGen Release 8 influenza GWAS summary statistics are available after accepting the terms and licenses. Influenza A seropositivity from Scepanovic et al., were downloaded from the GWAS catalogue (accession #GCST006339). Pre-calculated LD scores from the 1000 Genomes9 European reference population were obtained from https://data.broadinstitute.org/alkesgroup/LDSCORE/.

Human research participants

Policy information about studies involving human research participants and Sex and Gender in Research.

Reporting on sex and gender	Neither sex nor gender were considered in the study design. Analyses were not stratified by sex, although genetically determined sex was used as a covariate in the GWAS.
Population characteristics	Population characteristics (e.g., age, ancestry) can be found in Tables S1-2.
Recruitment	UK Biobank recruited approximately 500,000 individuals 40-69 years of age in 2006 to 2010 by mailers to people in the UK medical system (54.3% female). Informed consent was obtained for all participants. AncestryDNA customers over age 18, living in the United States, and who had consented to research, were invited to complete a survey assessing COVID-19 outcomes and other demographic information including SARS-CoV-2 swab and antibody test results, COVID-19 symptoms and severity, brief medical history, household and occupational exposure to SARS-CoV-2, and influenza infections (median age 57; 66.4% female). Geisinger Health System (GHS). The GHS MyCode Community Health Initiative is a health system-based cohort from central and eastern Pennsylvania (USA) with ongoing recruitment since 2006 (ages 19-94; 61.1% female). Penn Medicine BioBank (PMBB) study participants are recruited through the University of Pennsylvania Health System, which enrolls participants during hospital or clinic visits (ages 19-90; 50.7% female). Project Generation included 116,277 subjects from the Mayo Clinic Biobank (enrolled beginning in 2009) and 30 disease-specific registries (ages 19-98; 55.7% female). The ATLAS Precision Health Biobank at UCLA comprises ~32,000 individuals (ages 18-91; 55.7% female). De-identified phenotype data comprises all hospital visits beginning in 2013 and converted to ICD-10 codes. The CCPM biobank at the University of Colorado Anschutz Medical Campus in Aurora encompasses ~45,000 individuals (ages 30-92; 61.1% female). De-identified phenotype data was collected by Health Data Compass and comprises an individual's entire medical record from the University of Colorado's EHR.
Ethics oversight	Ethical approval for the UK Biobank was previously obtained from the North West Centre for Research Ethics Committee (11/NW/0382). The work described herein was approved by UK Biobank under application number 26041. GHS study: approval for DiscovEHR analyses was provided by the Geisinger Health System Institutional Review Board (#2006-0258). AncestryDNA study: all data for this research project was from subjects who provided prior informed consent to participate in AncestryDNA's Human Diversity Project, as reviewed and approved by our external IRB (Pro00034516), Advarra. All data was de-identified prior to use. PMBB study: appropriate consent was obtained from each participant regarding storage of biological specimens, genetic sequencing and genotyping, and access to all available EHR data. This study was approved by the Institutional Review Board of the University of Pennsylvania and complied with the principles set out in the Declaration of Helsinki. Mayo-RGC Project Generation: all subjects provided informed consent for use of specimens and data in genetic and health research and ethical approval for Project Generation was provided by the Mayo-Clinic IRB (#09-007763). CCPM Biobank: ethical approval and consent was reviewed and approved by the Colorado Multiple Institutional Review Board (#15-0461). UCLA: patient recruitment and sample collection for Precision Health Activities at UCLA is an approved study by the UCLA IRB (#17-001013). Informed consent was obtained for all study participants.

Note that full information on the approval of the study protocol must also be provided in the manuscript.

Field-specific reporting

Please select the one below that is the best fit for your research. If you are not sure, read the appropriate sections before making your selection.

☒ Life sciences ☐ Behavioural & social sciences ☐ Ecological, evolutionary & environmental sciences

For a reference copy of the document with all sections, see nature.com/documents/nr-reporting-summary-flat.pdf

Life sciences study design

All studies must disclose on these points even when the disclosure is negative.

Sample size	Sample sizes were all those available in the individual cohorts as described in the text. No power calculations were performed or required in advance.
Data exclusions	Prior to any analysis, we established the following data exclusions: We excluded individuals that were not predicted to belong to 5 continental ancestry groups (AFR, EAS, EUR, HLA, SAS) and furthermore did not analyze sets of individuals with fewer than 100 cases and 100 controls.
Replication	Of the 2 GWAS loci discovered in AncestryDNA, we successfully replicated both in a separate meta-analysis including FinnGen, UKB, UPENN-PMBB, GHS, Mayo Clinic, UCLA and Colorado. In vitro infection assays were successfully repeated at least twice.
Randomization	We performed a GWAS, which was an observational study, and as such no process of randomization was performed or applicable here because there was no allocation of samples into experimental groups.
Blinding	We performed a GWAS, which was an observational study, using coded de-identified data. As such, no process of blinding to group allocation was performed or applicable here.

Reporting for specific materials, systems and methods

We require information from authors about some types of materials, experimental systems and methods used in many studies. Here, indicate whether each material, system or method listed is relevant to your study. If you are not sure if a list item applies to your research, read the appropriate section before selecting a response.

Materials & experimental systems

n/a	Involved in the study	
☐	☒	Antibodies
☐	☒	Eukaryotic cell lines
☒	☐	Palaeontology and archaeology
☒	☐	Animals and other organisms
☒	☐	Clinical data
☒	☐	Dual use research of concern

Methods

n/a	Involved in the study	
☒	☐	ChIP-seq
☒	☐	Flow cytometry
☒	☐	MRI-based neuroimaging

Antibodies

Antibodies used	anti-ST6GAL1 antibody (goat, #AF5924, R&D system)<br>Chicken anti-goat HRP-conjugated antibody (#HAF019, R&D system)<br>Beta-actin HRP (#5123, Cell Signaling)<br>GAPDH HRP (#HRP-60004, Proteintech)
Validation	anti-ST6GAL1 antibody detects human ST6 Gal Sialyltransferase 1/ST6GAL1 in direct ELISAs and Western blots and has been cited in 23 publications. Both we and the vendor report reduction of the signal following siRNA-mediated knockdown. Chicken anti-goat HRP-conjugated antibody Detects goat IgG heavy and light chains in direct ELISAs and Western blots. In Western blots, less than 5% cross-reactivity with mouse IgG, rabbit IgG and human IgG is observed. It has been used in 23 publications. Both Beta-actin HRP and GAPDH HRP are common loading controls antibodies and they have been used in hundreds of publications.

Eukaryotic cell lines

Policy information about cell lines and Sex and Gender in Research

Cell line source(s)	A549 (CCL-185) and Calu-3 (HTB-55) cells were purchased from ATCC.
Authentication	Authentication via STR analysis was provided by the vendor (ATCC).

Mycoplasma contamination	Cultured cells were tested monthly for mycoplasma contamination using the Lonza MycoAlert Kit and tested negative.
Commonly misidentified lines<br>(See ICLAC register)	No commonly misidentified cells were used in this study.
