# Supplementary figures and images for: Genetic risk factors for COVID-19 and influenza are largely distinct
Source: Nat Genet. 2024 Aug 5;56(8):1592–6. doi: 10.1038/s41588-024-01844-1 (PMC11319199; doi:10.1038/s41588-024-01844-1)

a.

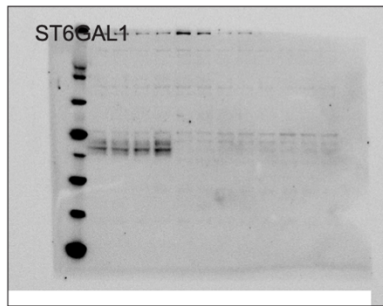

GAPDH

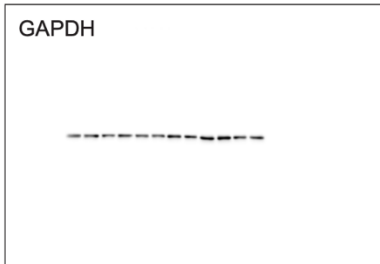

b.

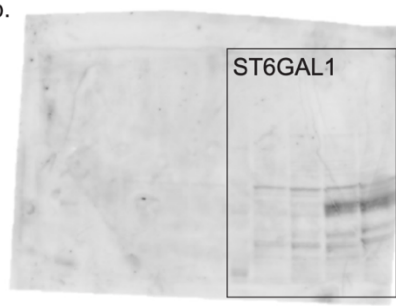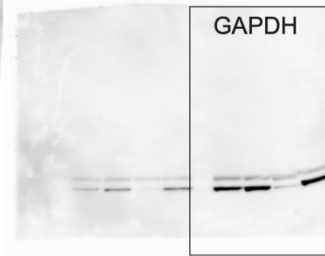

Beta Actin

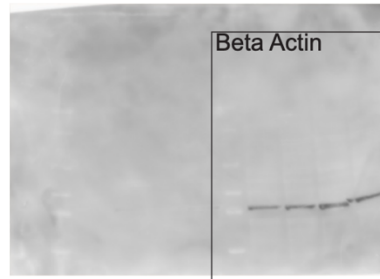

Supplement: Supplementary file 5 — Unprocessed immunoblots and gels. [file 41588_2024_1844_MOESM5_ESM.pdf]
